# Photoplethysmography-based non-invasive blood pressure monitoring via ensemble model and imbalanced dataset processing

**DOI:** 10.1007/s13246-024-01445-6

**Published:** 2024-07-30

**Authors:** Qianyu Liu, Chaojie Yang, Sen Yang, Chiew Foong Kwong, Jing Wang, Ning Zhou

**Affiliations:** 1 School of Information Science and Engineering, NingboTech University, Ningbo, China; 2 Department of Electrical and Electronic Engineering, University of Nottingham Ningbo, Ningbo, China; 3 Next Generation Internet of Everything (NGIoE) Laboratory, University of Nottingham Ningbo, Ningbo, China; 4 School of Civil Engineering and Architecture, NingboTech University, Ningbo, China; 5 Department of Electrical and Electronic Engineering, University of Nottingham, Nottingham, UK

**Keywords:** PPG, Machine learning, Hypertension, Data imbalance

## Abstract

Photoplethysmography, a widely embraced tool for non-invasive blood pressure (BP) monitoring, has demonstrated potential in BP prediction, especially when machine learning techniques are involved. However, predictions with a singular model often fall short in terms of accuracy. In order to counter this issue, we propose an innovative ensemble model that utilizes Light Gradient Boosting Machine (LightGBM) as the base estimator for predicting systolic and diastolic BP. This study included 115 women and 104 men, with experimental results indicating mean absolute errors of 5.63 mmHg and 9.36 mmHg for diastolic and systolic BP, in line with level B and C standards set by the British Hypertension Society. Additionally, our research confronts data imbalance in medical research which can detrimentally affect classification. Here we demonstrate an effective use for the Synthetic Minority Over-sampling Technique (SMOTE) with three nearest neighbors for handling moderate imbalanced datasets. The application of this method outperformed other methods in the field, achieving an F1 score of 81.6% and an AUC value of 0.895, emphasizing the potential value of SMOTE for addressing imbalanced datasets in medical research.

## Introduction

Blood pressure (BP) stands as a pivotal physiological metric in human health, serving as a cornerstone diagnostic indicator for various maladies, with hypertension emerging as a prominent exemplar. Hypertension is closely associated with the onset of cardiovascular disease and has been identified by the World Health Organization as a major contributor to global mortality rates [1]. Alarming statistics reveal a dismal reality where less than half of hypertensive cases receive accurate diagnoses, with even fewer achieving effective management [2]. Furthermore, a study conducted in 2016 elucidated the prevailing lackadaisical attitude towards BP monitoring, with a staggering 56% of participants measuring their BP sporadically, primarily relying on sporadic assessments from healthcare professionals [3].

### Background

In traditional medical practice, continuous BP monitoring relies on arterial line while it is an invasive method and not suitable for daily use. Cuff-based sphygmomanometer is another common non-invasive method. However, its restrictive nature impedes prolonged usage owing to its occlusive effect on blood flow dynamics [4]. This highlights the imperative for a novel paradigm in BP monitoring: a lightweight, wearable, and continuous non-invasive modality. Photoplethysmography (PPG) has emerged as a compelling frontier in this endeavor, offering not only enhanced convenience and flexibility over traditional monitors but also enabling seamless BP assessment during routine activities.

PPG signal, generated throughout the cardiac cycle, manifests as a pulsatile waveform reflecting the dynamics of cardiovascular hemodynamics [5]. Presently, the transmission method is widely employed to collect PPG signals, where the sensors are positioned on the fingertip or earlobe so that the light from the source passing through the tissue can be detected by a photodetector [5, 6]. The detection of PPG is a simple and cost-effective optical technique and only requires a small testing site on the skin, which makes it suitable for daily use and continuous monitoring.

### Literature review

PPG emerges as a promising non-invasive method for continuous BP estimation and potential detection of hypertension since it serves as a comprehensive representation of various physiological processes in the cardiovascular system. Several studies have demonstrated the effectiveness of PPG in BP field indicating though the precise biological correlation between BP and the PPG waveform remains not fully elucidated [7]. Traditional non-machine learning approaches such as Pulse Transit Time (PTT), Pulse Arrival Time (PAT) and Pulse Wave Velocity (PWV) [6] necessitate at least two synchronized sensors, with individual calibration requirements for each patient, thereby incurring additional time costs. In this study, Pulse Wave Analysis (PWA) is adopted as an advanced method, relying solely on a single PPG signal per patient, where features are extracted from the PPG waveform. The integration of machine learning techniques facilitates the optimization of models by utilizing a plethora of features, enabling the incorporation of other pertinent variables to a significant extent.

The main differences among different machine learning techniques lie in the models applied and the features used. However, it is also important to note that predictions across different databases cannot be directly compared due to differences in sampling techniques, types of sample objects, and overall sample size. Even for models using the same method, the results differs. There are existed models used for BP estimation such as linear regression (LR) [8], support vector machines (SVM) [8, 9], decision tree (DT) [10], DT based AdaBoost (AdaBoost-DT) [9], recurrent neural network (RNN) [11], convolutional neural network (CNN) [12], XgBoost [13]. Table 1 presents the prediction errors from these studies, each employing unique models and varying numbers of features. The comparison is based on mean absolute error (MAE) and standard deviation (STD).

**Table 1:** Comparison of previous studies

Researchers	Data Source	Feature type	Model	SBP (mmHg)		DBP (mmHg)	
				MAE	STD	MAE	STD
Zhang et al. [8]	Queensland	Feature	SVM	11.64	8.2	7.62	6.78
			Linear regression	12.22	10.72	9.2	7.06
Kachuee et al. [9]	MIMIC	Feature	SVM	12.26	10.32	5.91	5.78
			AdaBoost-DT	11.17	10.09	5.35	6.14
Shoeibi et al. [10]	MIMIC	Feature	DT	2.1	5.09	1.4	3.79
Aguirre et al. [11]	MIMIC	Raw signal	RNN (Seq2seq, Attention)	12.08	15.67	5.56	7.32
Schrumpf et al. [12]	MIMIC	Raw signal	CNN (ResNet, AlexNet)	13.4	10.1	8.27	4.67
Hu et al. [13]	3 databases	Feature	XgBoost	5.38	9.66	3.05	4.88
Ours	Scientifc data	Feature	AdaBoost-LGB	9.22	7.63	5.63	5.11

**Table 2:** Basic information of database and preprocessed dataset

Body information	Database		Preprocessed	
	Mean	STD	Mean	STD
Age (year)	57.17	15.87	57.42	15.98
Height (cm)	161.23	8.2	161.44	8.82
Weight (kg)	60.19	11.89	59.32	11.59
BMI (kg/m^2^)	23.11	4	22.83	3.93
Heart rate (bpm)	73.64	10.74	74.94	10.24
SBP (mmHg)	127.95	20.38	128.75	20.64
DBP (mmHg)	71.85	11.11	71.73	11.3

Although these models can solve BP estimation tasks to some extent, several challenges persist. Tree-structured models are prone to overfitting, sensitivity to noise, and may exhibit limited adaptability to data dynamics. SVM demands meticulous parameter tuning and are sensitive to outliers, potentially compromising their robustness. Moreover, ensemble models metioned above suffer either weak learners or protracted training periods. Neural networks, while powerful, may pose limitations concerning interpretability, training time, and suitability for tabular data.

In the context of BP monitoring, hypertension can be detected by calculating systolic BP (SBP) and diastolic BP (DBP) obtained from BP monitors, where it is defined when either SBP or DBP exceeds 130 mmHg or 80 mmHg, respectively. Recent research has focused on the hypertension detection as a classification problem, aiming to discern individuals with hypertension from normotensive counterparts. Patnaik et al. compared the performance of different classification models, including Naive Bayes (NB) classifiers, SVM, LR, random forest (RF), and multilayer perceptron (MLP) [14]. In their studies, SVM emerged as the most effective model, achieving the best AUC of 0.898 and accuracy of 80.23%. Liang et al. [15] conducted classification trials focusing on multiple domains and achieved the best results for hypertension classification with precision of 60% and F1 score of 71.19%. In a similar study, Tjahjadi et al. [16] used the *k*-nearest neighbors (KNN) algorithm for classification and achieved a better F1 score of 90.9% than Liang et al. [15].

On the other hand, despite the promising results, these studies often overlook the challenge posed by data imbalance between hypertensive and normotensive samples. Data imbalance is a common phenomenon in medical research where diseased instances are typically underrepresented. This imbalance can hinder the model’s ability to discern the minority class. Unfortunately, most methods for identifying hypertension do not thoroughly address this issue.

### Contribution

This paper utilizes PPG signals to estimate BP and detect potential hypertension based on standard clinical thresholds. In response to the issues mentioned above, this paper provides a more comprehensive feature engineering before proposing our advanced ensemble model. In face of the shortcoming of a single and weak ensemble models, we proposed a more advanced model AdaBoost-LGB. This model can effectively reduce overfitting and improve prediction efficiency and scalability. In addition to BP estimation, this paper also provides a detailed examination of the challenges posed by imbalanced datasets and proposes potential remedies for predicting skewed datasets. Following lists our main contributions:

- Comprehensive integration of features gleaned from disparate literature domains to distill salient predictors germane to BP estimation and hypertension classification.
- Development of an innovative ensemble learning framework, AdaBoost-LGB, amalgamating Adaptive Boosting as the core component with Light Gradient Boosting Machine (LightGBM) serving as the base estimator to transcend the limitations of singular models by enhancing robustness and scalability.
- Mitigation of data imbalance through judicious application of under-sampling and Synthetic Minority Over-sampling Technique (SMOTE), underpinning a more equitable classification paradigm for skewed datasets based on tree-structured classifier.

## Methodology

**Fig. 1:**
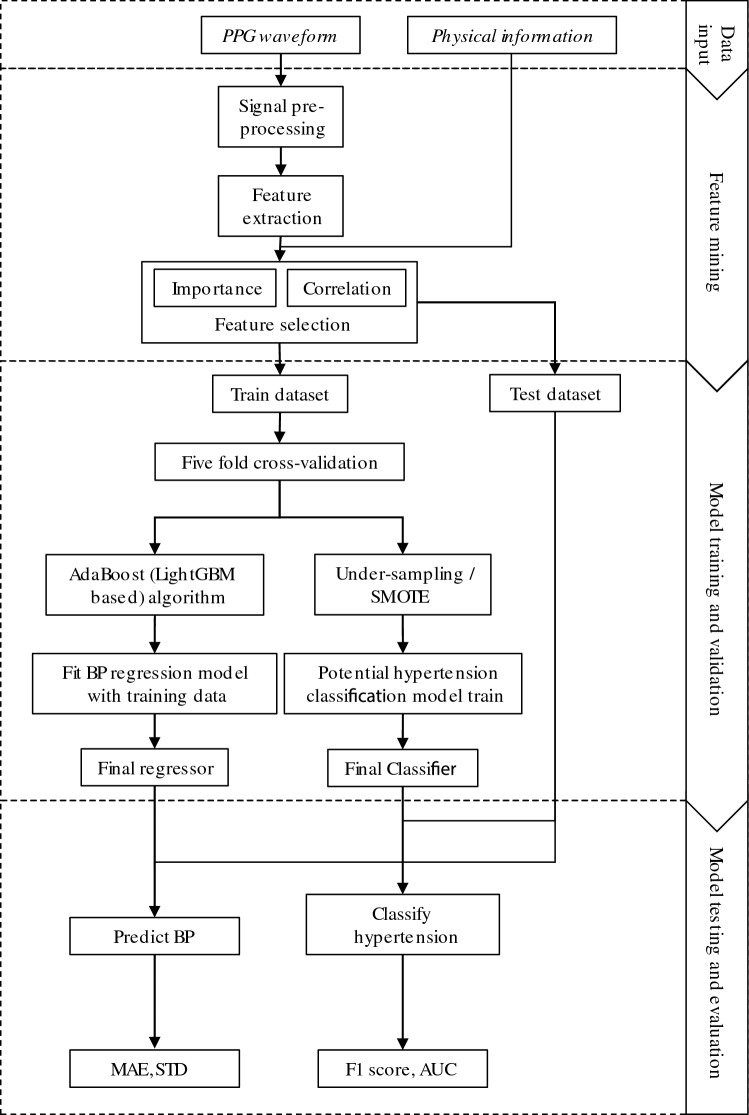
Block diagram of blood pressure (BP) prediction and classification of potential hypertension

This section describes the data acquisition, signal processing, feature extraction and selection, and models used in our study. The overall process is illustrated in Fig. 1.

### Database input and signal preprocessing

We obtained the dataset and original PPG signals from a database in Scientific Data [17]. The database consisted of PPG signals collected from the tip of the left index finger of 219 adult subjects using a portable hardware platform based on SEP9AF-2 (SMPLUS Company, Korea) PPG sensor, with a sampling frequency of 1 kHz and a conversion accuracy of 12 bits AD, and arterial blood pressure was measured from the right forearm simultaneously. During PPG collection, BP was also acquired using an Omron HEM-7201 device, which is a device intended for blood pressure self-measurement on the upper arm based on oscillometric technique [18]. Table 2 provides basic information about the participants. The database includes 115 female participants and 104 male participants; ‘mean’ shows the average of each set and ‘std’ shows their degrees of dispersion.

According to Liang et al. [17], they assessed the quality of the PPG signal by breaking the collection period three into segments, each lasting 2.1 seconds. A robust skewness-based signal quality index (S-SQI) method, which quantifies the symmetry in the shape of a probability distribution and is related to corrupted PPG signals, was used to evaluate each segment, where a higher S-SQI value indicated a higher signal quality. A segment normally contains 2-3 PPG cycles, which is suitable for PWA, as only one cycle is required to extract features. Longer segments were deemed unnecessary for this study and inefficient. A total of 657 PPG segments with S-SQI values were obtained. Each segment was individually assessed to determine its suitability for further analysis, with low-quality or incomplete-period segments discarded. Ultimately, 549 PPG waveform segments, along with the corresponding participant information, were chosen as the research materials for this study. The basic information of the preprocessed dataset is presented in Table 2. Among the selected segments, 53.55% belonged to male participants, ensuring a balanced gender representation.

**Table 3:** Abbreviations

Name	Abbr.
Photoplethysmography	PPG
Blood pressure	BP
Systolic blood pressure	SBP
Diastolic blood pressure	DBP
Pulse wave analysis	PWA
Skewness-based signal quality inde	S-SQI
Linear regression	LR
Decision tree	DT
Random forest	RF
Light gradient boosting machine	LightGBM
Synthetic minority over-sampling technique	SMOTE
Mean absolute error	MAE
Standard deviation	STD
Root mean square	RMS
Receiver operating characteristic	ROC
Area under the curve	AUC
True positive	TP
False negative	FN
False positive	FP
True negative	TN
False positive rate	FPR
True positive rate	TPR
Index of maximum slope	max_slope_idx
Value of maximum slope	max_slope_value
Systolic period	sys_period
Diastolic period	dia_period
Index of diastolic peak	dia_peak_idx
Value of diastolic peak	dia_peak_value
Fisher-Pearson coefficient	fisher
Width of 25, 50, 75% of pulse height at systolic stage	SW25, SW50, SW75
Width of 25% of pulse height at diastolic stage	DW25, DW50, DW75
Large artery stiffness index	LASI
Wavelet coefficient in level 1	cD1_max, CD1_min, CD1_mean, CD1_std
Wavelet coefficient in level 2	cD2_max, CD2_min, CD2_mean, CD2_std
Wavelet coefficient in level 3	cD3_max, CD3_min, CD3_mean, CD3_std
Wavelet coefficient in level 4	cD4_max, CD4_min, CD4_mean, CD4_std
Inflection point area ratio	s1, s2, s3, s4

**Table 4:** Model parameters in LightGBM, AdaBoost-DT, AdaBoost-LGB

	LightGBM	AdaBoost-DT	AdaBoost-LGB
Base_estimator	–	DT	LightGBM
Num_leaves	31	–	–
Reg_alpha	0.1	–	–
Objective	’regression’	–	–
Max_depth	− 1	–	–
Learning_rate	0.01	0.1	0.1
Min_child_samples	3	–	–
n_estimators	2000	300	300
Subsample	0.9	–	–
Colsample_bytree	0.7	–	–

**Table 5:** Predictions of different models

Feature type	Models	SBP		DBP	
		MAE (mmHg)	STD (mmHg)	MAE (mmHg)	STD (mmHg)
Demographic & PPG	LR	12.72	13.78	8.37	8.01
	DT	12.94	14.11	8.44	8.73
	LightGBM	9.36	7.92	5.64	5.39
	AdaBoost-DT	11.37	8.73	7.21	5.43
	AdaBoost-LGB	9.22	7.63	5.63	5.11
Demographic only	AdaBoost-LGB	14.25	12.03	9.86	8.06

**Table 6:** Classification abilities of different processing methods

	TP	TN	FN	FP	Recall (%)	Precision (%)	F1 score (%)	AUC
RF_Unprocessed	9	82	14	5	39.1	64.3	48.7	0.864
RF_Undersample	18	51	5	36	78.3	33.3	46.8	0.851
RF_SMOTE (k=3)	20	81	3	6	87.0	76.9	81.6	0.895
RF_SMOTE (k=5)	18	77	5	10	78.3	64.3	70.6	0.888
RF_SMOTE (k=10)	16	68	7	19	69.6	45.7	55.2	0.875
Regression	13	87	10	0	56.5	100	72.2	–

**Table 7:** Evaluation by BHS standard

		$\le 5 mmHg (\%)$	$\le 10 mmHg (\%)$	$\le 15 mmHg (\%)$
Our results	SBP	40	66.4	88.3
	DBP	59.1	80.9	92.8
BHS grade	A	60	85	95
	B	50	75	90
	C	40	65	85
	D	Worse than C		

To identify potential hypertension in participants, standard clinical criteria were employed: SBP higher than 130 mmHg or DBP greater than 80 mmHg. In the preprocessed dataset derived from the database of [17], 19.67% of the subjects (108 participants) were identified as potential hypertension.

A Fast Fourier Transform (FFT) was performed on several PPG signals in order to find a suitable and common cut-off frequency range for the bandpass Butterworth filter to filter out the noise. The filtered signals were then normalized to have unit variance on a scale from zero to one.

### Dataset split

In this study, the dataset was initially split into two subsets: a training set and a testing set, in a proportion of 0.8 to 0.2. To address the issue of overfitting in traditional models, where the model is overly trained on the training set and hence performs poorly on the testing set, a five-fold cross-validation technique was employed on the training set. Specifically, the training set was divided into fivefolds, with each fold serving as a validation set while the remaining four folds acted as the training set. This process was repeated 5 times, ensuring that each fold was utilized as a validation set once. Consequently, five models were trained and applied to the testing set. The final error was obtained by averaging the predictions generated by the five cross-validated models.

### Feature extraction

The features used in this study can be categorized into physical information and PPG waveform morphology, and their names and abbreviations are given in Table 3. As mentioned in "PPG signal processing" Section, the physical characteristics included age, height, weight, gender, and BMI. Additionally, features describing the morphology of the PPG waveform were extracted, as they are deemed important predictors based on the theory of waveform morphology.

Firstly, various basic waveform features were calculated, including period, mean, STD, RMS, max_slope_idx, max_slope_value, as well as systolic and diastolic information such as sys_period and dia_period, dia_peak_idx, dia_peak_value. Additionally, the Fisher-Pearson coefficient (fisher) of segment skewness was calculated as shown in Eq. 1 [19].

1 $$\begin{aligned} g=\frac{\sum _{i=1}^{n}\left( T_i-{\bar{T}}\right) ^3}{ns^3} \end{aligned}$$

where $T_i$ represents the value of each point in a period, $\bar{T}$ is the average of all $T_i$ in a period, *s* is the standard deviation, and *n* is the total number of points in a period.

**Fig. 2:**
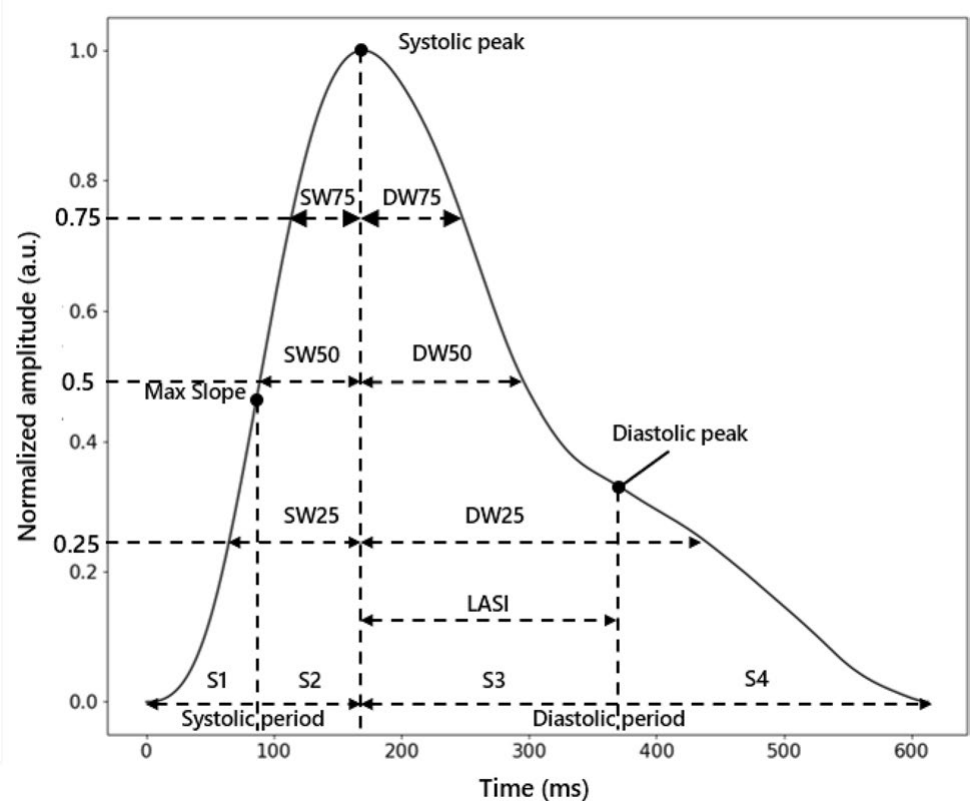
Time domain features of PPG singal in one cycle (x-axis represents time in ms, y-axis represents normalized amplitude to [0,1] with arbitrary unit)

Secondly, several pulse durations were selected as features. The width of 25%, 50% and 75% of total height were measured from the diastolic and systolic stage of the PPG waveforms [8]. The ratio of systolic duration and diastolic duration at the same height was then added. The Large Artery Stiffness Index (LASI) was also considered to be an important feature and extracted from the time difference between the systolic and diastolic peaks [9]. Figure 2 illustrates some features in the time domain.

Thirdly, Wu et al. advised that wavelet transform (WT) can be accessed as a feature extraction method [20]. WT is a local transformation of time and frequency can perform multiscale detailed analysis of signals. In this study, multilevel 1D Discrete WT was used to process discrete PPG signal with the decomposition level of 4, and the wavelet coefficient vectors were returned in 4 different levels: level 1 (cD1), level 2 (cD2), level 3 (cD3) and level 4 (CA4, cD4), which represents detail ponderances of 0-0.3125 Hz, 0.3125-0.625 Hz, 0.625-1.25 Hz, 1.25-2.5 Hz and 2.5-5 Hz, respectively. To simplify these vectors, the maximum, minimum, mean and STD values of each vector were added as features.

Finally, amplitude features were added for prediction, including Augmentation (ratio between the height of the diastolic peak and the systolic peak) and Inflection Point Area ratio (IPA, areas under the PPG curve separated by the max slope at systolic stage, systolic peak and diastolic peak - s1, s2, s3 and s4) [9]. In total, 48 features were extracted.

### Feature selection

**Fig. 3:**
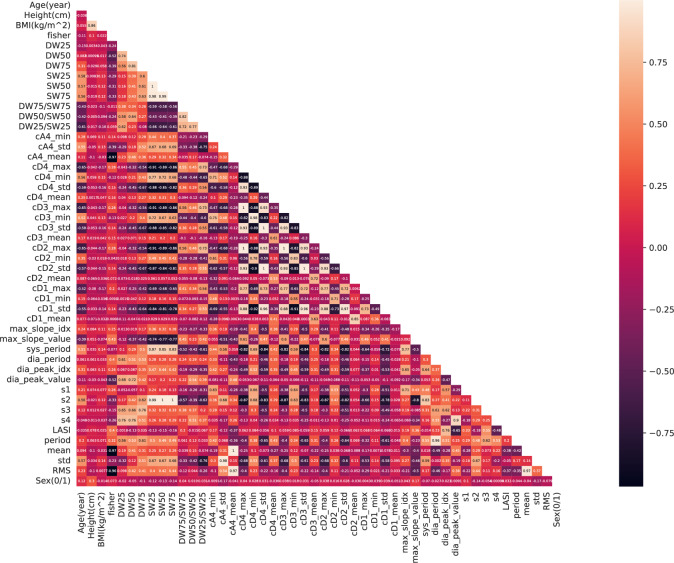
Correlation of extracted features

Correlation and importance analyses were used as featuring ranking criteria. Initially, a heat map was generated to assess the correlation among different features. As shown in Fig. 3, several features extracted from WT exhibit high internal similarity, such as cD4_max, cD3_max, cD3_max, and cD2_max, and high correlation with SW25, SW50, and SW75. Additionally, s2 is highly related to pulse duration on the systolic side, as it corresponds to that specific area. Overall, there was minimal correlation between physical features and PPG morphological features. However, some morphological features showed non-negligible correlations with each other, as mentioned earlier. Therefore, it was deemed optimal to select one of two strongly correlated features. For instance, s2 can be replaced by sys_period, and SW25 can be replaced by SW50, among others.

**Fig. 4:**
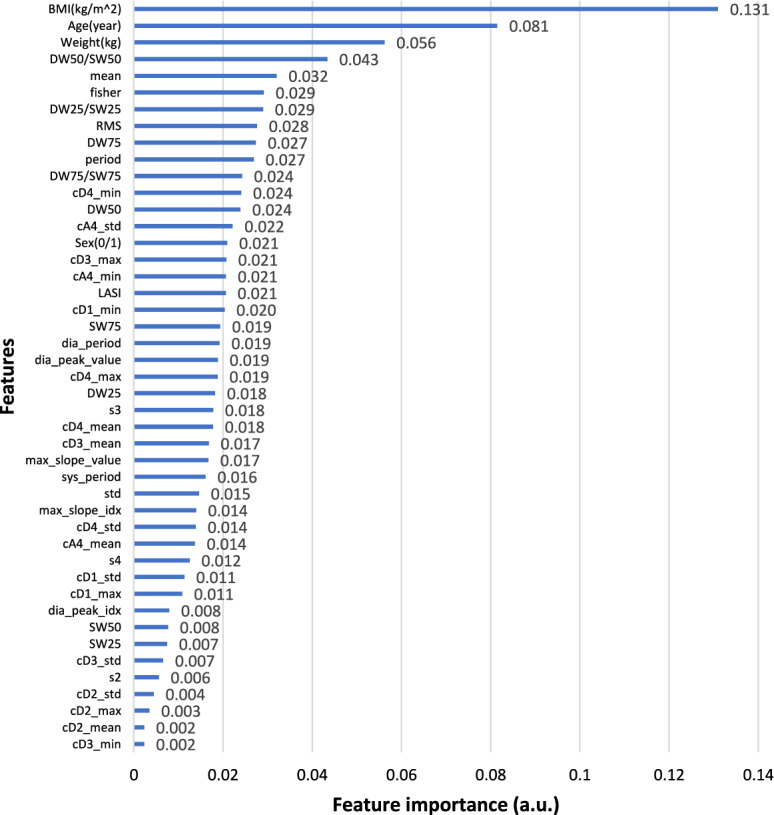
Gini importance of features in SBP prediction

**Fig. 5:**
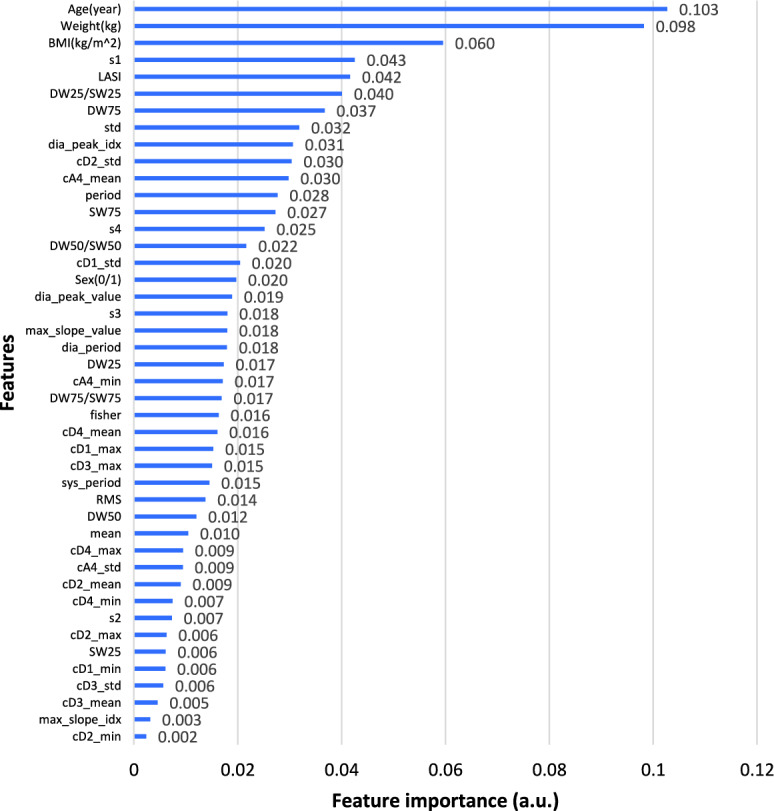
Gini importance of features in DBP prediction

After conducting the correlation analysis, additional feature selection approach of importance analysis were employed to determine whether and to what extent the features had an impact on the results, where DT was used to provide a quantitative analysis of feature ranking, offering a numerical measure of feature importance. Figures 4 and 5 depict the non-zero contributions of the various extracted features to the prediction of SBP and DBP. Feature importance is measured by calculating the total decrease of the criterion resulting from each feature, also known as the Gini importance. The equation for calculating the weighted impurity decrease, which represents the feature importance, is shown in Eq. 2. In this equation, *N* represents the total number of samples, $N_t$ represents the number of samples at the current node, $N_{t,L}$ represents the number of samples in the left child, and $N_{t,R}$ represents the number of samples in the right child. *G* is the Gini index at the current node, while $G_L$ and $G_R$ are the Gini indices of the left child and the right child, respectively. A higher feature importance value indicates a more significant feature.

2 $$\begin{aligned} Feature\ Importance=\frac{N_t}{N}\times \left( G-\frac{N_{t,R}}{N_t}G_R-\frac{N_{t,L}}{N_t}G_L\right) \end{aligned}$$

Considering both the feature importance ranking and correlation analysis, it was concluded that physical features, excluding gender, were crucial for prediction. Additionally, diastolic duration at total height of 25% and 75%, LASI, information at cA4, cD3, cD2, and diastolic and systolic periods were selected as features for predicting SBP and DBP.

**Fig. 6:**
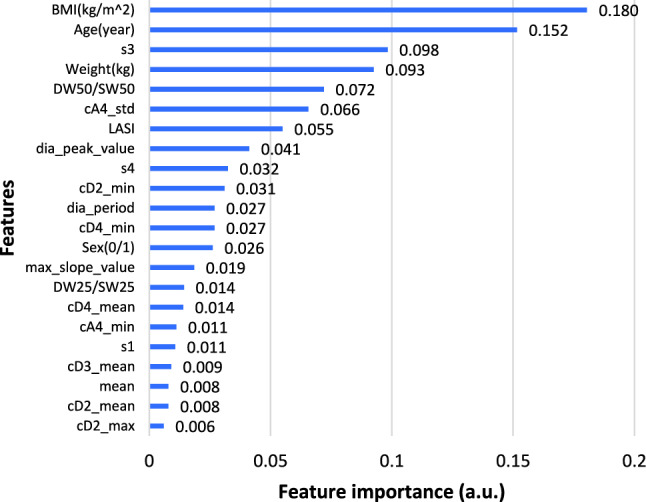
Gini importance of features in hypertension classification

Similarly, Gini importance was also applied to analyze features in classifying potential hypertension. Features with non-zero contributions to classification are as shown in Fig. 6. Therefore, features used for classification were physical features including BMI, age, gender and weight, and signal features like s3, diastolic duration at total height of 25% and 50%, LASI, information at cA4, cD4, cD2, s1, s3, s4, diastolic peak and period.

### Regression model

In regression models, LR was set as the benchmark model since it was the simplest linear model and could be used as a baseline. Then, DT, AdaBoost-DT and LightGBM were trained separately. These models were used to compare with the proposed ensemble model, AdaBoost-LGB. DT is a basic model based on a tree structure, which consists of numerous nodes and branches at every node [21]. Also, it is the default base estimator in AdaBoost. LightGBM implements several optimization techniques for training and prediction [22].

**Fig. 7:**
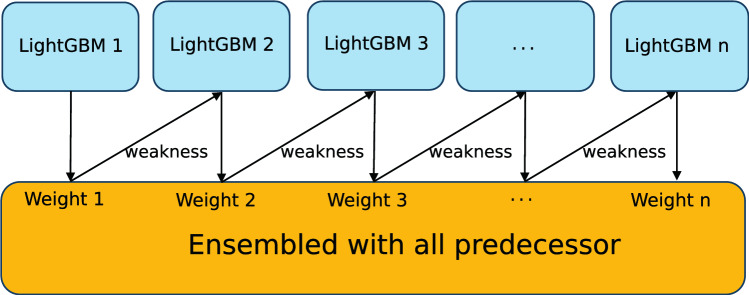
Boosting diagram with LightGBM as the base model

In this study, an ensemble model was used as the core algorithm because it solves the problem that a single model is not ideal for partial prediction results. Algorithm 1 presents the pseudo-code for AdaBoost-LGB. Firstly, *T*-round iterations and the weak learner (LightGBM) are set. Secondly, equal weights ($D_t$) are initialized to each training example. Within iteration rounds, the model is trained in each iteration and $D_t$ of the current weak learner is updated with the loss function ($L_t(i)$) as:

3 $$\begin{aligned} D_{t+1}(i)=\frac{ D_t(i)\beta ^{1-L_t(i)}}{Z_t} \end{aligned}$$

where $Z_t$ is a normalization factor chosen so that $D_{t+1}$ will be a distribution. When the average loss or the round number exceed the upper limit, iteration will be stopped and the output of the different learners will be combined to produce the BP estimation, which is the weighted median of the weak learners’ results. Figure 7 shows the visualized process of boosting using LightGBM as the base model. As mentioned above, the iteration stops at the round limit or average loss.

**Algorithm 1:**
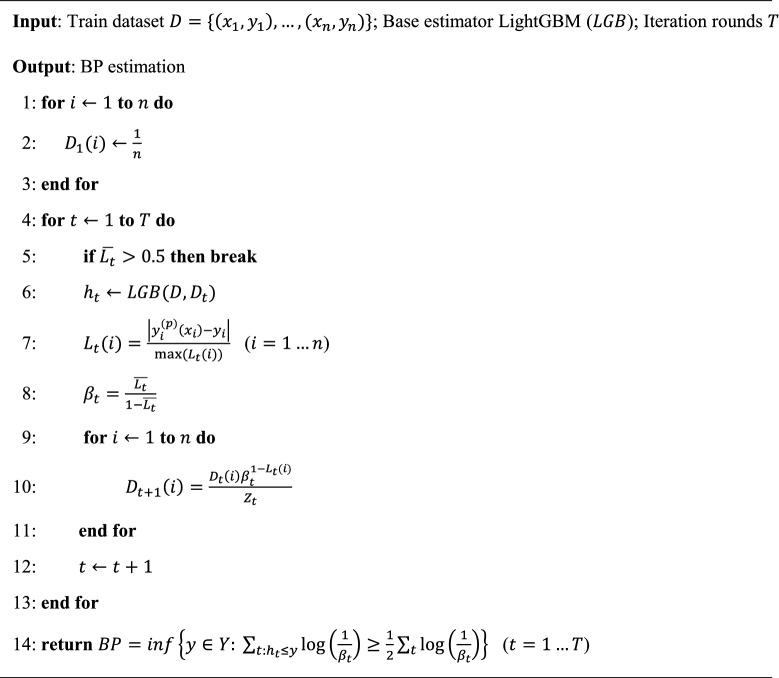
AdaBoost Regression with base estimator as LightGBM

All models were achieved by Python. The parameters in AdaBoost-LGB remained the same as in single-use models to control for the variables, except for the default base estimator in AdaBoost, which was DT. Table 4 presents the parameters in AdaBoost-DT, LightGBM, and AdaBoost-LGB. Other models retained their default parameters. Overall, AdaBoost-LGB was the experimental group while LR, DT, AB, and LightGBM were the control groups.

### Imbalanced data handling and classification

In this study, the proportion of the minority class, the potential hypertension group, was about 19.67% (Positive:Negative = 108:441), which belongs to a moderate imbalance. This study compared the following methods for dealing with an imbalanced dataset: under-sampling and SMOTE with different *k*-nearest neighbors.

1. Under-sampling: This method is the simplest approach for dealing with an imbalanced dataset. It randomly removes a subset of the majority class to equalize its number to the minority class. In this dataset, the negative samples are randomly eliminated to 108.
2. SMOTE: SMOTE is one of the most popular data sampling algorithms among oversampling techniques. To apply SMOTE on this dataset, firstly, for each sample X in the minority class, the distance to all samples in the minority class sample set is calculated using the Euclidean distance *d*(*p*, *q*) as the standard with Eq. 4, and the *k*-nearest neighbors are obtained, where $(p_{1} ,q_{1} )$ and $(p_{2} ,q_{2} )$ are the positions of two samples.
  4 $$d(p,q) = \sqrt {(p_{1} - q_{1} )^{2} + (p_{2} - q_{2} )^{2} } {\text{ }}$$

Then, a sampling fraction corresponding to the imbalanced proportion of the samples is set to determine the sampling ratio n. For each minority sample x, several samples are randomly selected from their *k*-nearest neighbors, assuming that the nearest neighbor selected is $x_n$. For each randomly selected nearest neighbor $x_n$, a new sample is constructed with the original sample according to the following Eq. 5.

5 $$\begin{aligned} x_{new} = x + rand\left( {0,~1} \right) \times \left( {x_{n} - x} \right) \end{aligned}$$

For the classification of underlying high hypertension, the control group was the unprocessed imbalanced dataset, while the experimental groups were the dataset with imbalance handling: under-sampling, SMOTE with $k=3, 5, 10$.

In addition to handling the imbalanced dataset, RF was chosen as the training model for classification. It has accuracy unmatched among current algorithms and is based on the ensemble learning method of bagging, while extending the applicable scope. RF therefore does not require complex tuning and is therefore suitable as a model for comparing classification predictions.

## Results

### PPG signal processing

**Fig. 8:**
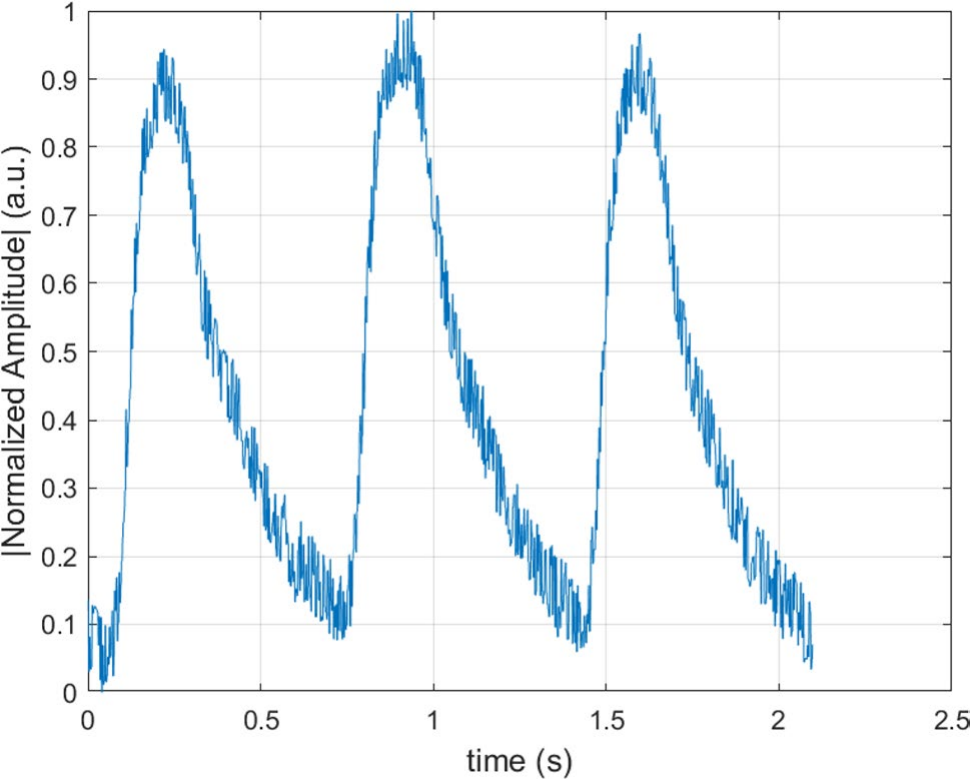
Normalized raw PPG segment example [17]

**Fig. 9:**
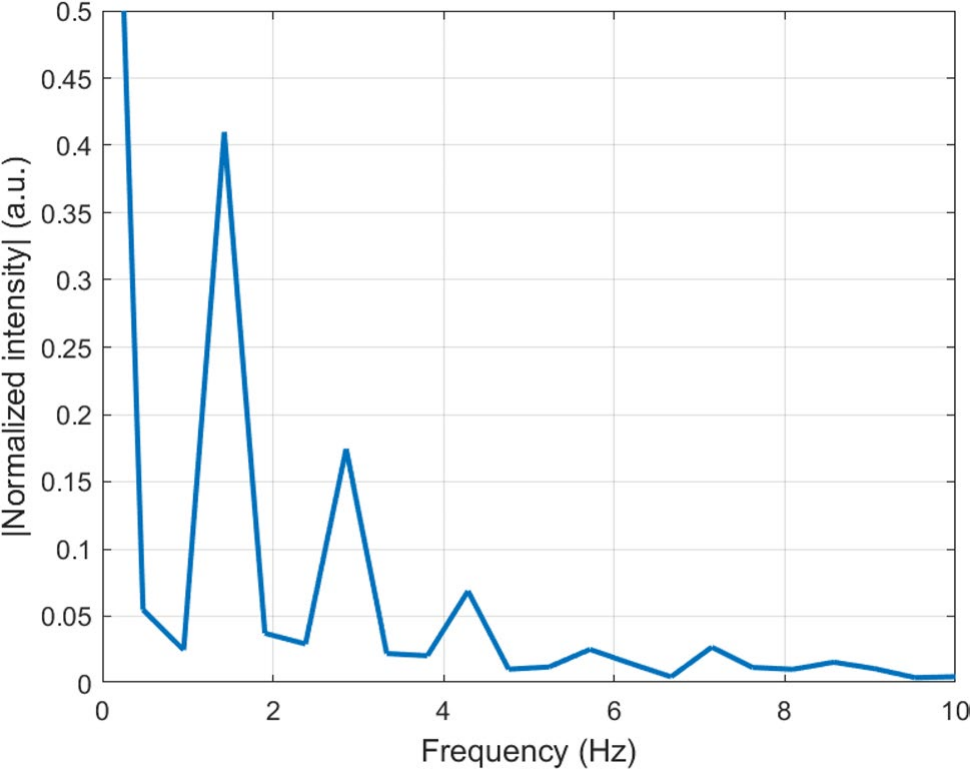
FFT of raw PPG signal

Figure 8 was the example segment PPG signal obtained from [17] with ID number 125, which has been normalized, and Fig. 9 was the FFT of this segment. Except the high DC component, the first peak around 1.5 Hz was normally considered as the heart rate frequency and the peaks around 3 Hz and 4.5 Hz indicated the harmonics. Therefore, a band pass Butterworh filter was chosen with an order of 6 and frequency range of [0.5,5Hz].

### BP prediction

As for BP regression, the estimation results were evaluated using MAE between predicted and measured values. Meanwhile, STD was calculated from absolute errors to show the dispersion degree of these errors. $BP^{(p)}_{i}$ is the predicted BP while $BP_{i}$ is measured BP; $\overline{BP^{(p)}}$ is the average of all predicted BP values and *n* is the total number of samples.

6 $$\begin{aligned}{} & {} MAE = \frac{\sum _{i = 1}^{n}\left| {{BP^{(p)}_{i}} - BP_{i}} \right| }{n} \end{aligned}$$

7 $$\begin{aligned}{} & {} STD = \sqrt{\frac{ {\textstyle \sum _{i=1}^{n}\left| BP^{(p)}_i-\overline{BP^{(p)}} \right| } }{n-1}} \end{aligned}$$

Table 5 shows the prediction results of AdaBoost-LGB compared to control models. Notably, our proposed model, AdaBoost-LGB, coupled with demographic features, emerges as the frontrunner in terms of predictive accuracy, boasting the lowest MAE and STD across both SBP and DBP estimation tasks. Specifically, AdaBoost-LGB achieved an impressive MAE of 9.22 mmHg and STD of 7.63 mmHg for SBP estimation, and an equally commendable MAE of 5.63 mmHg and STD of 5.11 mmHg for DBP estimation. These results underscore the robustness and efficacy of our proposed methodology in accurately predicting BP values.

While LightGBM exhibited the second-best performance, the disparities in prediction accuracy between AdaBoost-LGB and the control models, including LR, DT and AdaBoost-DT, are stark. This proves the effectiveness of the proposed model. These findings affirm the superior predictive capability of AdaBoost-LGB.

With only demographic features, AdaBoost_LGB yielded less favorable results, manifesting in a MAE of 14.25 mmHg and a STD of 12.03 mmHg for SBP estimation, and a MAE of 9.86 mmHg and a STD of 8.06 mmHg for DBP estimation, even worse than the baseline model LR, which indicates the effectiveness of PPG signal on BP estimation.

### Classification of potential hypertension

When classifying an imbalanced dataset, commonly used evaluation methods, such as accuracy, may not be valid in most circumstances. This is because in imbalanced cases, the training result tends to favor the majority class, leading to high accuracy but potentially failing to identify the minority class. In this study, the model was evaluated using the receiver operating characteristic (ROC) curve, including the area under the curve (AUC) value, as well as the confusion matrix and related metrics such as precision, recall, and F1 score.

The x-axis of the ROC curve represents the False Positive Rate (FPR, 1-specifity), and the y-axis represents the True Positive Rate (TPR, sensitivity). The ROC curve is an effective method for evaluating classifiers in imbalanced datasets. A no skill classifier would have an AUC value of 0.5, while a perfect classifier would have an AUC value of 1. The ROC curve is particularly suitable for moderate imbalances as seen in this study. In detection, sensitivity represents the ability of the model to predict positive samples correctly, i.e., the proportion of true positives (TP) correctly identified by the model. Specificity represents the ability of the model to predict negative samples correctly, i.e., the proportion of true negatives (TN) correctly identified by the model. From the perspective of prediction results, precision is the rate of correctly predicted positive samples out of all positive samples predicted by the binary classifier. Recall, which is numerically the same as sensitivity, represents the ability to recall real positive samples from the test set, indicating the rate of real positive samples recalled by the classifier. Equations 8, 9, 10 and 11 show the mathematical calculation of sensitivity, specificity, precision, recall, and F1 score, where TP is true positive, FP is false positive, FN is false negative, and TN is true negative - the four values in the confusion matrix [23].

8 $$\begin{aligned}{} & {} sensitivity = \frac{TP}{TP + FN} \end{aligned}$$

9 $$\begin{aligned}{} & {} specifity = \frac{TN}{FP + TN} \end{aligned}$$

10 $$\begin{aligned}{} & {} precision = \frac{TP}{TP + FP} \end{aligned}$$

11 $$\begin{aligned}{} & {} recall = TPR = \frac{TP}{TP + FN} \end{aligned}$$

Precision and recall are related to each other and can sometimes be inverted. When a model has a high precision, it means that most of its positive predictions are correct while it may miss some relevant items (low recall). On the contrary, when a model has high recall, it means that it identifies most of the relevant items while it may also include some false positives (low precision). Therefore, F1 score is applied to find a balance between recall and precision, which is calculated as Eq. 12.

12 $$\begin{aligned} F1~score = 2 \times \frac{precision \times recall~}{precision + recall} \end{aligned}$$

Two assessment indicators for classification methods, F1 score and AUC, have the same standard, which is recall (TPR), and they aim to train a model that fits the sample data well. In addition to recall, another parameter is precision, which the F1 score focuses on. The goal of this indicator is to find as many usable models as possible. In contrast, AUC focuses on true negative rate (TNR) so that a conservatively error-free model is trained. Therefore, the F1 score, especially recall, should be considered first to create a model that detects all cases, and then AUC is used to evaluate a very accurate architecture.

**Fig. 10:**
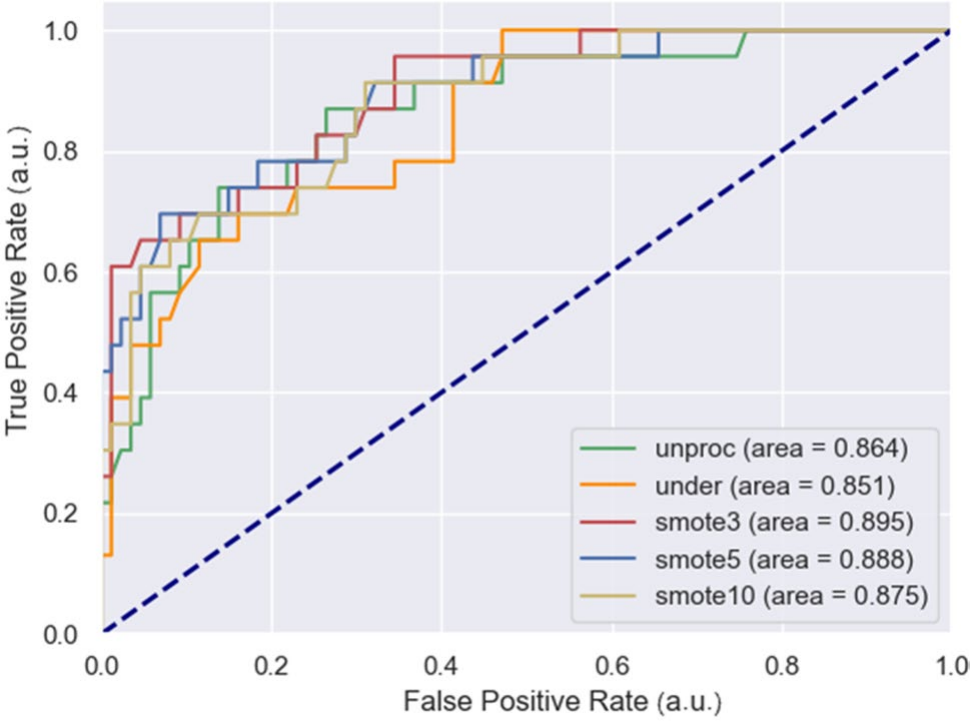
ROC curves of hypertension classification with unprocessed, undersampled, SMOTE processed (different k values) dataset

The performance of the different methods used for classification is presented in Table 6, along with information related to the confusion matrix. The ROC curves of these classification methods are displayed in Fig. 10.

Among these methods, SMOTE, with *k*-nearest neighbors equal to 3, achieved the best classification results, as evidenced by both the ROC curves and F1 score. This configuration of SMOTE had the largest AUC of 0.895, indicating that it was a relatively accurate model, and its high F1 score of 81.6% demonstrated its effectiveness. The high recall of 87% suggests that this method is possible to find out almost all hypertension samples while the relatively poor precision of 76.9% illustrates somewhat over-searching. Conversely, using under-sampling to handle imbalanced data resulted in poor prediction results with AUC of 0.851 and F1 score of 46.8%, even worse than classification with unprocessed imbalanced dataset (48.7% F1 score & 0.864 AUC). In addition to classification prediction, the identification of potential hypertension from estimated BP values predicted by AdaBoost-LGB led to a medium F1 score of 72.2%. The high precision and low recall suggest that the predicted values were generally lower than the actual values, resulting in more false negative (FN) samples. When blood pressure is first predicted and then used to identify hypertension, the results have high precision but low recall.

## Discussion

The comparative analysis of various training models provides valuable insights into their efficacy in BP estimation. AdaBoost-LGB emerged as the most proficient model, showcasing superior MAE and STD values when compared to control models, excelling in predicting both SBP and DBP when leveraging demographic features. Notably, LightGBM also performed well, with almost the same MAE as AdaBoost-LGB. However, DT had the largest estimation errors in predicting SBP and DBP among the tested models, and performed even worse than LR.

As a contrastive view, AdaBoost-LGB was also used to train a model only with demographic features, and the result was found to be worse than the models trained with demographic and PPG features.The inclusion of PPG features yielded discernibly superior results, underscoring the discernible influence of PPG signals on BP estimation accuracy.

Table 7 presents the results of AdaBoost-LGB, evaluated using the British Hypertension Society (BHS) protocol. The BHS protocol assesses the performance of BP estimation or measurement systems based on the cumulative percentage of absolute errors between the estimated values and the actual measured values at three different thresholds (i.e., 5 mmHg, 10 mmHg, and 15 mmHg) [24]. As shown in Table 7, the proposed estimator in our study, AdaBoost-LGB, achieved level C in SBP prediction and level B in DBP prediction. Comparing SBP prediction with DBP prediction, the former was relatively worse than the latter. This is because the basic value of SBP is larger than that of DBP, and this greater cardinality can lead to a larger error. Our BP estimation results also compared with the literature in Table 1. The results outperform deep learning methods using raw signal data and feature-based models like LR, SVM, AdaBoost-DT while cannot exceed some other methods. Despite disparities in sample composition, these comparisons underscore the promising trajectory of our model.

Among these training models, AdaBoost-LGB was proven to be the most effective model, with superior MAE and STD values. This also indicates that LightGBM plays an important role in improving the performance of the ensemble model as a base estimator. Its strength lies in advanced optimization techniques. Compared to LR, it is able to capture complex nonlinear relationship effectively attributed to its leaf-wise tree growth and histogram-based splitting. Growing trees in a leaf-wise manner, it chooses the leaf with the maximum reduction in loss to split at each level. This approach leads to deeper trees compared to the level-wise splitting strategy used in traditional DT, resulting in improved predictive performance. Additionally, hyperparamters for regularization play a pivotal role in averting the perils of overfitting commonly encountered with DT models, which reflects on parameter reg_alpha in Table 4. reg_alpha works as L1 regularization that penalizes large coefficients so that it helps prevent overfitting by discouraging complex models. In addition to leaf-wise tree growth, LightGBM uses a histogram-based approach for finding the best split points during tree construction, which can reduce the computational cost and improve scalability. These salient features collectively underpin the effectiveness of LightGBM as a robust and versatile weak learner, distinguishing it from the performance limitations inherent in DT.

Comparative analysis between AdaBoost-LGB and AdaBoost-DT elucidates the disparity in their performance outcomes. The subpar performance of DT can be attributed to their susceptibility to overfitting and poor generalization, rendering them ill-suited for integration within AdaBoost frameworks. Conversely, AdaBoost-LGB capitalizes on LightGBM’s inherent strengths, leveraging deeper trees, robustness against overfitting, and enhanced efficiency in hyperparameter tuning to propel performance gains. This strategic amalgamation not only mitigates the performance deficiencies associated with DT models but also affords AdaBoost-LGB a competitive edge in terms of predictive accuracy and generalization capacity.

Working as a ensemble model, AdaBoost-LGB not only take advantageous of LightGBM including resistance to overfitting and improved predictive performance, but also utilizes multiple weak learners (LightGBM) to form a stronger ensemble model. Compared to common ensmble models such as Bagging and Stacking, AdaBoost, a popular algorithm in Boosting, is a powerful ensemble modeling method that trains weak learners in parallel, using the previous model’s errors to train the next model. It is sensitive to outliers, so it can, to a large extent, perform additional training on those outlier samples to improve prediction performance and avoid overfitting. Therefore, AdaBoost-LGB obtains better prediction than LightGBM alone by integrating AdaBoost’s ensemble learning principles with the powerful optimization techniques of LightGBM.

In the results of classification, detection with an unprocessed imbalanced dataset leads to a poor F1 score but high AUC. Although this hypertension classifier has good adaptability to the change in thresholds, it performs poorly in terms of precision and recall, indicating that it struggles to identify instances correctly of the positive class and minimize FP and FN classes. Classification with undersampled dataset results to even worse poor precision and F1 score than imbalanced dataset possibly because of a large amount of missing data. On the other hand, when classifying potential hypertension, high recall is preferable to high precision because failure to identify underlying hypertension may lead to higher costs. It demonstrates the feasibility of model ensemble. Hence, undersampling is more preferable than unprocessed dataset in some situation.

It can be concluded that SMOTE with *k*-nearest neighbors equal to three is a suitable method for processing this imbalanced hypertension dataset. It increased the minority class in the positive sample set (potential hypertension class) by inserting new samples between existing adjacent samples, so that both sides of the classification could be equally considered. However, when the value of *k* increases, two distant samples could become linked, resulting in the newly created sample falling into the domain that should have been a negative sample. This is the reason why $k=5, 10$ has poorer results. When the degree of imbalance increases, or the minority class has an extremely limited number of samples, another option is to use a one-class classifier by scaling the majority class to distinguish it from the minority class [25].

It is imperative to acknowledge the inherent trade-offs associated with each approach. While an unprocessed dataset may yield suboptimal F1 scores, undersampling risks data loss and potential underfitting, whereas SMOTE supplementation introduces the risk of generating synthetic samples at incorrect locations with inappropriate *k* values. Therefore, meticulous consideration of *k* value selection is imperative to ensure the efficacy and integrity of the classification process.

In summary, AdaBoost-LGB can serve as a valuable tool for continuous BP monitoring with PPG signal, enabling healthcare practitioners to obtain real-time insights into patients’ cardiovascular health. Additionally, PWA technique, which is used in this study, employed necessitates only a single PPG signal, obviating the need for multiple sensors and facilitating seamless integration into wearable devices for daily BP monitoring. This streamlined approach holds significant promise for enhancing patient compliance and facilitating proactive management of hypertension. Furthermore, the accurate classification of hypertension is paramount in identifying individuals at risk and instituting timely interventions. This paper underscores the challenges posed by imbalanced datasets in training hypertension classifiers and highlights SMOTE as an effective strategy for mitigating classification bias. By rectifying imbalances and ensuring equitable consideration of both positive and negative instances, SMOTE enhances the robustness and accuracy of hypertension classification models.

In essence, the integration of AdaBoost-LGB with PPG signals presents a transformative opportunity to revolutionize BP monitoring practices, empowering both healthcare practitioners and individuals alike with actionable insights into cardiovascular health. Furthermore, by addressing the challenges associated with imbalanced datasets, this methodology lays the groundwork for the development of more accurate and reliable hypertension classifiers, thus advancing the forefront of preventive healthcare and personalized medicine.

## Conclusion

In this study, the ensemble model, with LightGBM as the base estimator, was shown to be efficient in BP prediction. Moreover, it is confirmed that an imbalanced dataset has a serious negative impact on classification, and SMOTE can effectively improve this with an appropriate k value. The proposed method uses signal preprocessing, RFE to search for dominant features, a novel model AdaBoost-LGB for regression, and imbalanced dataset analysis for classification. Compared with the BHS standard, the enhanced model achieves grade B in DBP prediction and grade C in SBP prediction. In the results for identification of underlying hypertension, the F1 score reached as high as 81.6% with AUC of 0.895.

As a novel model, AdaBoost-LGB met the prediction expectation. Much work could be done on the PPG waveform processing to further improve the prediction accuracy, which we address here. It will be beneficial for future work to classify different types of PPG signals with different features and prediction methods. In addition, a novel prediction method without feature extraction could be attempted by inputting PPG signals as image information for predicting via CNN. Also, more samples could be collected in the future for more comprehensive prediction.
